# The effect of spinal manipulative therapy and home stretching exercises on heart rate variability in patients with persistent or recurrent neck pain: a randomized controlled trial

**DOI:** 10.1186/s12998-021-00406-0

**Published:** 2021-11-29

**Authors:** Anders Galaasen Bakken, Andreas Eklund, David M. Hallman, Iben Axén

**Affiliations:** 1 Department of Environmental Medicine, Division of Intervention and Implementation Research for Worker Health, Karolinska Institutet, Nobels väg 13, 171 77 Stockholm, Sweden; 2 Centre for Musculoskeletal Research (CBF), Department of Occupational Health Sciences and Psychology, University of Gävle, Gävle, Sweden

**Keywords:** Manipulative therapy, Stretching exercises, Heart Rate Variability, HRV, Autonomic nervous system

## Abstract

**Background:**

Persistent or recurrent neck pain is, together with other chronic conditions, suggested to be associated with disturbances of the Autonomic Nervous System. Acute effects on the Autonomic Nervous System, commonly measured using Heart Rate Variability, have been observed with manual therapy. This study aimed to investigate the effect on Heart Rate Variability in (1) a combination of home stretching exercises and spinal manipulative therapy versus (2) home stretching exercises alone over 2 weeks in participants with persistent or recurrent neck pain.

**Methods:**

A randomized controlled clinical trial was carried out in five multidisciplinary primary care clinics in Stockholm from January 2019 to April 2020. The study sample consisted of 131 participants with a history of persistent or recurrent neck. All participants performed home stretching exercises daily for 2 weeks and were scheduled for four treatments during this period, with the intervention group receiving spinal manipulative therapy in addition to the home exercises. Heart Rate Variability at rest was measured at baseline, after 1 week, and after 2 weeks, with RMSSD (Root mean square of successive RR interval differences) as the primary outcome. Both groups were blinded to the other group intervention. Thus, they were aware of the purpose of the trial but not the details of the “other” intervention. The researchers collecting data were blinded to treatment allocation, as was the statistician performing data analyses. The clinicians provided treatment for participants in both groups and could not be blinded. A linear mixed-effects model with continuous variables and person-specific random intercept was used to investigate the group-time interaction using an intention to treat analysis.

**Results:**

Sixty-six participants were randomized to the intervention group and sixty-five to the control group. For RMSSD, a B coefficient of 0.4 (*p* value: 0.9) was found, indicating a non-significant difference in the regression slope for each time point with the control group as reference. No statistically significant differences were found between groups for any of the Heart Rate Variability indices.

**Conclusion:**

Adding four treatments of spinal manipulation therapy to a 2-week program of daily stretching exercises gave no significant change in Heart Rate Variability.

*Trial Registration*: The trial was registered 03/07/2018 at ClinicalTrials.gov, registration number: NCT03576846. (https://pubmed.ncbi.nlm.nih.gov/31606042/)

**Supplementary Information:**

The online version contains supplementary material available at 10.1186/s12998-021-00406-0.

## Introduction

Chronic pain affects people globally and is estimated to be the reason for 15% to 20% of all physician visits [1]. Chronic neck pain (NP) was globally ranked fifth in 2015 measured by years lived with disability [2]. In 2017, the worldwide prevalence of neck pain was found to be 3551 per 100.000 [3].”

Chronic NP is, like all types of chronic pain, a complicated matter. The discussion has reached a point where the term “chronic pain” is now questioned, and other definitions have been suggested [4, 5]. This study uses persistent or recurrent pain synonymously for chronic pain, because studies have shown that chronic pain varies in intensity and most often is episodic [4, 5].

The etiology of NP is not fully understood, but some factors contributing to the development of persistent or recurrent NP have been identified. An initial trauma can trigger acute pain episodes transitioning into chronic NP [6]. Degenerative changes have also been suggested as an underlying cause [7, 8]. However, tissue damage does not have to be present for chronic NP to develop [9]. Psychological factors, such as emotional trauma [10] or distress [11, 12], are also associated with the etiology of chronic NP. Signs of central sensitization and reduced inhibitory mechanisms [13] are often found among these patients, indicating increased sympathetic activation and reduced parasympathetic activation [14, 15]. These two branches make up the Autonomic Nervous System (ANS) [16] and are functionally and anatomically distinct. They are responsible for maintaining homeostasis by regulating cells, tissues, and the function of organs. The ANS is regulated by supraspinal centers such as the limbic system, hypothalamus, and some brainstem nuclei, particularly the periaqueductal gray area [16].

Sympathetic and parasympathetic nervous system activity is altered in chronic pain conditions such as chronic low back and neck-shoulder pain, fibromyalgia, complex regional pain syndrome, and phantom limb pain [17, 18].

Disruption in autonomic balance can be measured using Heart Rate Variability (HRV) [17]. This is a marker of the vagal components of the heart’s sinus node and measures the beat-to-beat changes in intervals [19, 20]. In general, a high HRV indicates a well-functioning and adaptable ANS, while a low HRV indicates a poor-functioning ANS and is associated with a range of poor health outcomes [17, 21–23].

A range of treatment options exists for chronic NP [24]. One commonly used treatment method for musculoskeletal pain is Spinal Manipulative Therapy (SMT), defined as High-Velocity low Amplitude thrusts or mobilization of the spinal joints [25]. This technique is used by a range of professions [26] and has been shown to be effective for the treatment of NP, especially in combination with exercise therapy [27–29]. Based on a limited number of studies, the mechanisms of the pain-reducing effect of SMT proposed by Bialosky et al. [30] are thought to be multifactorial.

Several systematic reviews of the acute effects of SMT on ANS have been conducted [31–35]. However, these systematic reviews investigate a variety of SMT techniques and ANS outcome measures [34]. Only one systematic review includes assessment of risk of bias and evaluation of the quality of the outcome measures [34]. When summarizing these results, an acute (immediately after the intervention) ANS response can be observed with most SMT techniques. The evidence is of very low to moderate quality [31–35]. The exact mechanism of this immediate effect is not known.

There is a lack of well controlled Randomized Controlled Trials (RCT) investigating the long-term effects of SMT on HRV. The present study design was seen as the natural next step of the investigation into SMT and HRV. Two weeks was chosen and defined as long-term, based on previous research in the area investigating immediate effects. Because this study investigates patients potentially seeking care at a clinic, a pure placebo trial was not indicated [36]. Home stretching exercises were chosen as the comparison group because it was a viable treatment option for patients with neck pain. However, on the basis of previous research and current guidelines [24, 27, 37, 38], it was expected to have a smaller effect on pain and HRV than a combination of home stretching exercises and SMT. Stretching exercises are commonly used together with strengthening exercises and have been found to have a pain-reducing effect on persistent or recurrent NP, whilst home stretching exercises alone have been found to have a small or no beneficial effect [37]. In a study of women with chronic NP, the pain-reducing effect of stretching was similar to that of manual therapy alone [39].

The mechanism behind the pain-reducing effects of stretching is thought to be reduced neuronal discharge by inhibition of Golgi tendon organs [40]. Acute changes in the tension-length relationship in muscle tissue lead to greater flexibility, affected by the individual stretch tolerance [41–45] and possibly changes in the muscle’s viscoelasticity [46]. Acute increases in HRV have also been seen with stretching exercises [47–51].

As recurrent or persistent NP is associated with imbalanced autonomic activity, e.g. reduced HRV, it is important to identify whether recommended treatments aimed at improving pain, such as SMT in combination with stretching exercises, also restore ANS balance in this patient group.

We hypothesized that four treatments with SMT and home stretching exercises are more effective in improving HRV than home stretching exercises alone.

This study aimed to investigate the effects of a 2-week treatment series consisting of (1) home stretching exercises and SMT versus (2) home stretching exercises alone on HRV in a population of patients with recurrent or persistent NP.

## Method

The study is reported according to the CONSORT statement guidelines.

This was a multicenter study carried out in five multi-professional clinics within the regional health service in Stockholm, Sweden. Clinics were chosen based on being similar in terms geographical location, having multi professional teams and subsidization of treatments. Chiropractors, dieticians, occupational therapists, and physiotherapists worked at all these clinics. All chiropractors were licensed by the Swedish National Board of Health and Welfare.

Recruitment began in January 2019, with the data collection ending in April 2020. The final follow-up questionnaires were answered in June 2020.

This article is the second publication reporting on the outcomes from an RCT, described in a published protocol [25] which provides detailed information about the study procedure and method.

### Recruitment

Participants were recruited from patients seeking care at the participating clinics and collaborating GP clinics as well as advertisements in clinics’ newsletters, Facebook, and local newspapers. The recruitment channels were adapted to fit the local procedures at each clinic. All the participants were screened for eligibility over the phone using a standardized check list and booked in for five treatments at the clinic by the primary researcher.

### Inclusion criteria

- Presence of recurrent (at least one previous episode) and persistent (duration more than 6  months) NP [52].
- No chiropractic treatment in the previous 3 months.
- Minimum 18 years of age
- Able to read and write Swedish

### Exclusion criteria

Conditions or medications that could affect the HRV measurements, such as

- diagnosed with cardiovascular disease
- diagnosed with hypertension
- diagnosed with diabetes type I or II
- pregnancy
- obesity (BMI > 30)
- on steroid medication
- on β-blocker medication
- on antidepressant medication

Participants were also excluded if they had

- serious, competing diagnoses such as cancer, infection, or recent severe trauma
- contra-indications to spinal manipulation, e.g. the recent development of headache or dizziness, previous drop-attacks, or acute cervical radiculopathy.

### Randomization

SPSS version 20 (https://spss.software.informer.com/20.0/) was used to generate randomly permuted blocks of different sizes using a 1:1 allocation ratio by a research assistant. The same research assistant also prepared consecutively numbered sealed opaque envelopes containing participant information and group allocation. The sealed envelope was brought to the treating chiropractor at the first visit and opened there, providing the allocated treatment modality.

### Blinding

The clinicians participating in the study were not blinded, as they were delivering the treatments. The researchers collecting the data were blinded to group allocation, and the main analysis was carried out by a statistician also blinded to group allocation.

The participants did not know what treatment the other group was receiving. They were told that both groups were receiving treatments commonly used for persistent or recurrent NP. Thus, they were aware of the purpose of the trial but not the details of the “other” intervention. All participants (both treatment arms) received the same examination, support, and opportunity to ask questions about their condition. Treatment adherence was essential in both groups to ensure effective delivery of both interventions.

### Intervention

All participants were booked for five visits to the clinic. The intervention included the four treatments and ended after the final measurements before the fifth visit. All visits had at least 2 days between them, with a maximum of two treatments each week for all participants.

One group received home stretching exercises (Additional file 5) and SMT (intervention group); the other received home stretching exercises only (control group). All clinicians provided treatments in both groups.

*Intervention group* On the basis of patient preferences and clinical impressions such as physical function and palpatory findings, the chiropractor tailored the type of SMT to the individual subject while conforming to the study's definition of SMT, i.e. High-Velocity low Amplitude thrusts or mobilization to the spinal joints [25]. Any area of the spine could be treated to allow for an individualized clinical approach by the clinicians, but also to allow for treatment when participants were reluctant to have treatment performed to the painful neck itself. Also, systematic reviews [31–35] have not been able to conclude on whether short-term sympathetic upregulation found with SMT is related to the spinal area being treated.

*Control group* The control group received the same examinations and verbal information as the intervention group for all visits, excluding any passive treatment. Thus, they discussed their exercises and pain with their chiropractor on subsequent visits.

All participants were asked to keep an exercise diary to monitor adherence to the home stretching exercises. Information from the patient files were obtained after the study period ended to control the adherence to SMT.

### Baseline

During the first visit to the clinic (i.e. baseline), participants received written information about the study's purpose, data protection, contact information, and information about the follow-up questionnaires and daily SMS message. A consent form was signed before any measurements took place. They were then asked to fill in their first questionnaires on-site. This included demographics and questions concerning their recent caffeine and alcohol consumption, medication intake, and recent exercise.

After this, participants were placed in a quiet room with hearing protection. The first 5 min were then used as relaxation time to reduce any effect on HRV from external factors. During the next 5 min, the HRV measurement was recorded in a resting state. The participants were then randomized and met their chiropractor for the initial consultation and first treatment session.

### Follow-up

The participants were measured again prior to the third and fifth visits to the clinic (i.e. 1 and 2 weeks after the first treatment) for a total of 2 weeks. This was done to prevent any acute effect from treatment and ensure a standardized time interval between measurements. Three weekly follow up measurements of HRV were chosen to include sufficient amount of data without requiring too much time from the participants. At follow-up, the HRV measurement was recorded with the same procedure and conditions as at baseline.

### Outcome

#### Heart rate variability at rest

HRV is recognized as a valid and reliable non-invasive measure of ANS and can be regarded as a biomarker for ANS regulation [17, 53]. It has excellent reliability of indices reflecting central parasympathetic control over the heart rate either on a frequency- or time-domain analysis [54]. HRV at rest has been found to have moderate test–retest reliability in healthy adults [55]. HRV during standardized rest reflects resting autonomic cardiac modulation and is a valuable outcome in interventions targeting ANS in patients with pain [56]. Earlier research on HRV and persistent or recurrent NP have also used this strategy [52]. We measured HRV at rest for 5 min. This is a standard short-term recording described by the Task Force Standards [56].

A heart rate monitor (Bodyguard2, Firstbeat Technologies Oy, Jyväskylä, Finland) was used to record R-R intervals at rest using a standard 2-lead ECG configuration (https://www.firstbeat.com/en/). This is a small portable device that is attached to the chest with Kendal Arbo H92SG electrodes. The device measures R-R intervals with a sampling rate of 1000 Hz. The time series of R-R intervals were stored directly on the device and downloaded to a PC for off-line analysis of resting HRV. The Bodyguard2 monitor has been found to produce results for indices of heart rate variability that are similar to the gold-standard laboratory electrocardiogram (Biopac MP150) during resting conditions [57].

The Taskforce of the European Society of Cardiology and the North American Society of Pacing and Electrophysiology [56] has developed standards of measurements to be used when investigating HRV. These were slightly adapted on the basis of previous research [58], the indices are summarized in Table 1.

**Table 1:** Heart Rate Variability indices suggested by The Taskforce of the European Society of Cardiology and the North American Society of Pacing and Electrophysiology

HRV indices	Indicator of	Domain measure	Change that improves HRV
R-R interval	Global HRV activity	Time	Increase
Root mean squared successive differences between IBIs (RMSSD)	Parasympathetic (vagal) activity	Time	Increase
The standard deviation of IBIs (SDNN)	Global HRV	Time	Increase
Low frequency power (LF, 0.04–0.15 Hz)	Baroreceptor-sympathetic and parasympathetic cardiac activity	Frequency	Increase
High frequency power (HF, 0.15–0.4 Hz)	Parasympathetic (vagal) activity	Frequency	Increase
LF/HF ratio	Sympathetic-to-parasympathetic balance	Frequency	Decrease
Total power	Global HRV activity	Frequency	Increase

All indices are expected to increase with improved HRV except for the LF/HF ratio

#### Data processing of HRV

Prior to HRV analysis, all recordings of R-R intervals were visually inspected and manually cleaned for ectopic beats and artifacts by the primary researcher blinded to the treatment allocation, using Kubios software [59]. Both frequency domain and time parameters were analyzed using 5-min segments. Threshold-based beat correction algorithm testing with different sensitivity filters of R-R intervals was used. Several sensitivity filters exist in the software, ranging from 0.45 to 0.05 s differing from the local sample average [59]. Five-minute periods with more than 5% artifacts were excluded. This is in line with a previous study [60]. In this study, 6.7% of all analyzed data were excluded due to measurement error. Group difference on pain and disability in this study is reported in a separate article [61].

#### Adverse reactions

The participants were asked to report any adverse reactions from the first treatment by SMS message after their first visit, with an NRS-11 scale anchored by the descriptors ‘No reaction’ (0) and ‘Worst reaction imaginable’ (10) [62].

#### Sample size

Sample size was calculated a priori based on the outcome lnRMSSD, which is the primary time-domain measure, minimally affected by respiration [63]. The sample size was calculated based on values obtained from the article by Hallman et al. [52], and logarithmic values where used as we had reliable data on the means and distributions of the logarithmic values and wanted to base the study size on reliable information. A difference of 10–20% in logRMSSD has been considered clinically important [64]. Sixty participants were needed in each treatment arm to reach a power of 80% with a significant level of 5% to detect a change in lnRMSSD of 10% between groups [65].

#### Ethics

Stretching and SMT are considered to be safe and effective treatments for NP [66].

A unique number was assigned to each study subject by a research assistant at the time of inclusion. A code key linking the participant's unique number and ID is stored according to the Swedish National Board of Health and Welfare's requirements for the safekeeping of medical records.

#### Statistical analysis

**Fig. 1:**
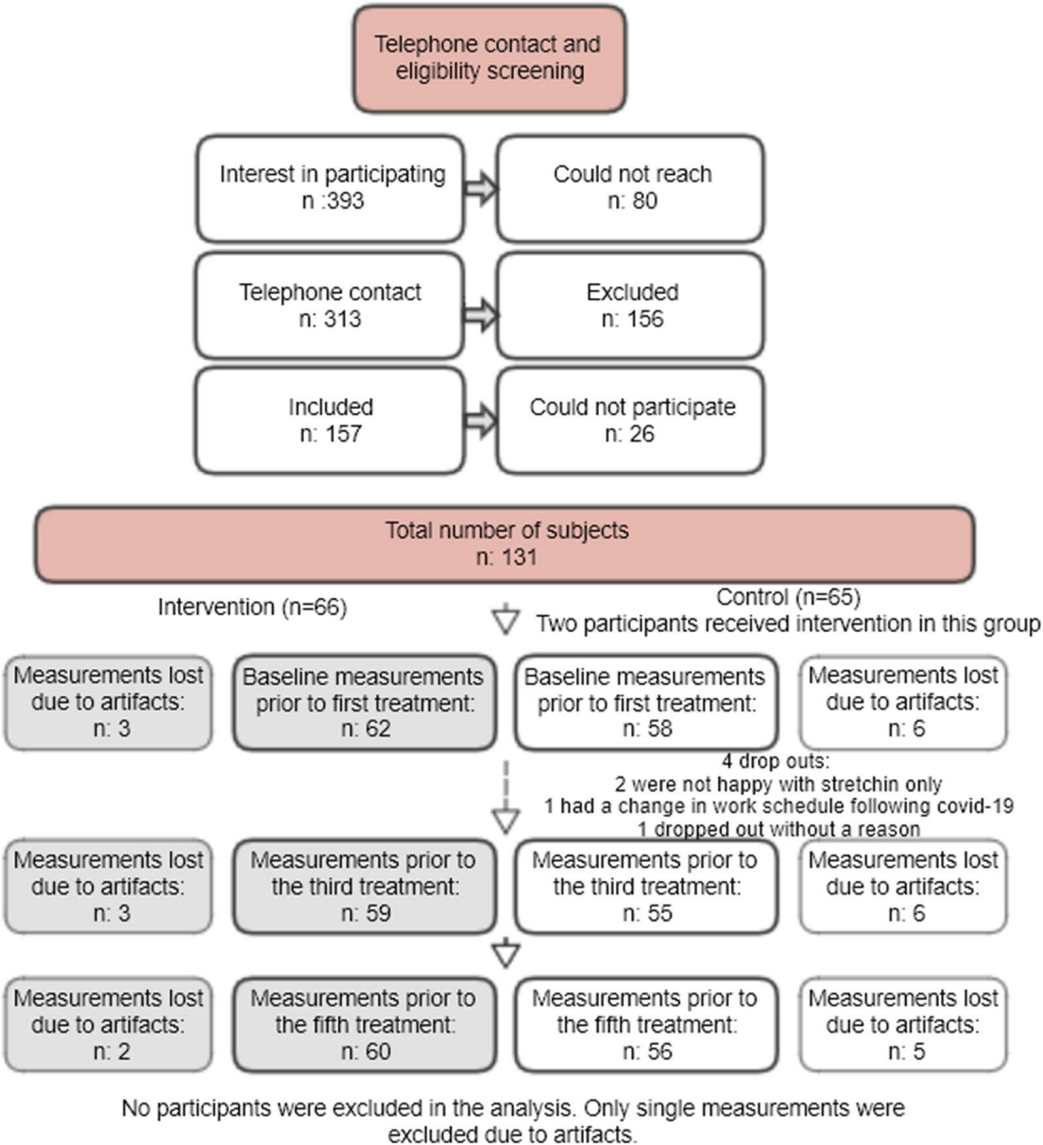
Flow chart of the study measurements with exclusions. Number of subjects measured at each time point differs slightly due to procedural errors or missed appointments

Intention to treat analysis was used. Missing values were not imputed as only 11.8% of all observations were missing. These included dropouts, measurement error, and missed appointments, as reported in Fig. 1. A per protocol analysis was conducted as a sensitivity analysis to account for dropouts and group allocation mix ups.

The intervention effect on HRV indices (outcome) was analyzed using linear mixed-effects models with group, time, and the interaction as fixed effects, and a person specific random intercept. This interaction between group allocation and time can be interpreted as the difference in the groups’ regression line for each time-point (1 and 2 weeks).

An additional analysis was performed, adjusting for age, and gender as stated in the protocol. The effect of baseline pain intensity on the changes in HRV is not known, thus baseline differences in pain intensity were also adjusted for. All terms were entered at once.

Outliers were investigated with a sensitivity analysis, excluding all outliers visually disproportionally distant to the mean, but this analysis did not significantly affect the results.

A per-protocol analysis was also performed, which followed the same method as the primary analysis with repeated measures. This did not significantly affect the results.

The time effect for the total study population was calculated using a separate linear mixed-effects model without adding group allocation. This was done to investigate the change of the entire group because no group difference was observed. *P* values smaller than 0.05 were considered significant. The analysis was performed using SPSS 27 [67] and Stata version 15 (StataCorp. 2017).

The graphical representations were done using a linear regression model representing the estimated difference between groups over 2 weeks.

## Results

### Baseline

A total of 131 patients were included in the study, 66 in the intervention group and 65 in the control group. After cleaning the HRV data, 25/350 measurements were lost due to insufficient ECG quality. See the flow chart (Fig. 1) for details.

The groups were similar at baseline, except for mean NP intensity (NRS-11), which was slightly higher for the intervention group, reported in Table 2. This difference is not considered clinically relevant [68].

**Table 2:** Demographics of the study population at baseline, n = 131

	Intervention (66)	Control (65)
Age, mean (sd)	57 (14.0)	58 (13.7)
Female, n (%)	37 (56)	36 (55)
Baseline neck pain NRS-11, mean	4.68	4.17
Arm pain, n (%)	42 (65)	36 (57)
Pain in the midback, n (%)	39 (61)	37 (62)
Pain in the low back, n (%)	39 (62)	37 (59)
*Neck pain*		
1. Less than 6 months, n (%)	0 (0)	1 (2)
2. 6–12 months, n (%)	8 (12)	10 (16)
3. Several years, n (%)	57 (88)	51 (82)
*STarT back categories*		
1. Low risk, n (%)	47 (80)	48 (79)
2. Medium risk, n (%)	7 (12)	11 (18)
3. High risk, n (%)	5 (9)	2 (3)
*Sick leave during previous year*		
Does not work, n (%)	13 (20)	18 (28)
No, n (%)	47 (71)	41 (63)
Yes, between 1 and 7 days, n (%)	3 (5)	2 (3)
Yes, between 8 and 14 days, n (%)	3 (5)	0 (0)
Yes, more than 15 days, n (%)	0 (0)	4 (6)

Some differences between the groups at baseline for the HRV outcome measures were also seen. HRV indices were slightly higher in the intervention group, reported in Table 3 together with mean differences.

**Table 3:** Means and mean differences of all indices of heart rate variability at rest (n = 123)

	Intervention (n = 62)		Control (n = 61)	
	Mean	SD	Mean	SD
*Mean R-R interval (ms)*				
BL	843	129	887	138
1 week	828	107	894	122
2 weeks	831	145	882	155
2 weeks-BL	− 16	105	− 21	97
*Root mean square of successive differences (RMSSD) (ms)*				
BL	27	29	29	21
1 week	24	21	26	21
2 weeks	25	28	26	16
2 weeks-BL	− 3	16	− 4	18
*Standard deviation of normal to normal (SDNN) (ms)*				
BL	27	18	32	18
1 week	26	15	29	18
2 weeks	25	17	29	17
BL-2 weeks	− 2	12	− 4	14
*Low frequency (LF) (ms*^*2*^*)*				
BL	361	475	700	955
1 week	365	498	623	935
2 weeks	355	736	616	1434
BL-2 weeks	− 5	455	− 106	1051
*High frequency (HF) (ms*^*2*^*)*				
BL	329	569	347	449
1 week	304	449	371	535
2 weeks	249	292	294	357
BL-2 weeks	− 86	475	− 70	445
*Low frequency/high frequency ratio (LF/HF)*				
BL	2.6	4.0	2.7	2.4
1 week	2.5	2.6	2.4	2.1
2 weeks	3.1	4.7	2.7	3.5
BL-2 weeks	0.5	4.9	0.2	4.0
*Total power (ms*^*2*^*)*				
BL	741	862	1133	1302
1 week	706	722	1065	1406
2 weeks	628	893	988	1678
BL-2 weeks	− 117	803	− 185	1351

Log and absolute values did not differ in precision or direction compared to the absolute values and yielded the same conclusions. The absolute values are presented in this article as they are easier to interpret and reported in similar research [31, 69, 70]. The results from the analysis of log values are found in Additional file 4.

### Intervention effect on heart rate variability at rest

**Fig. 2:**
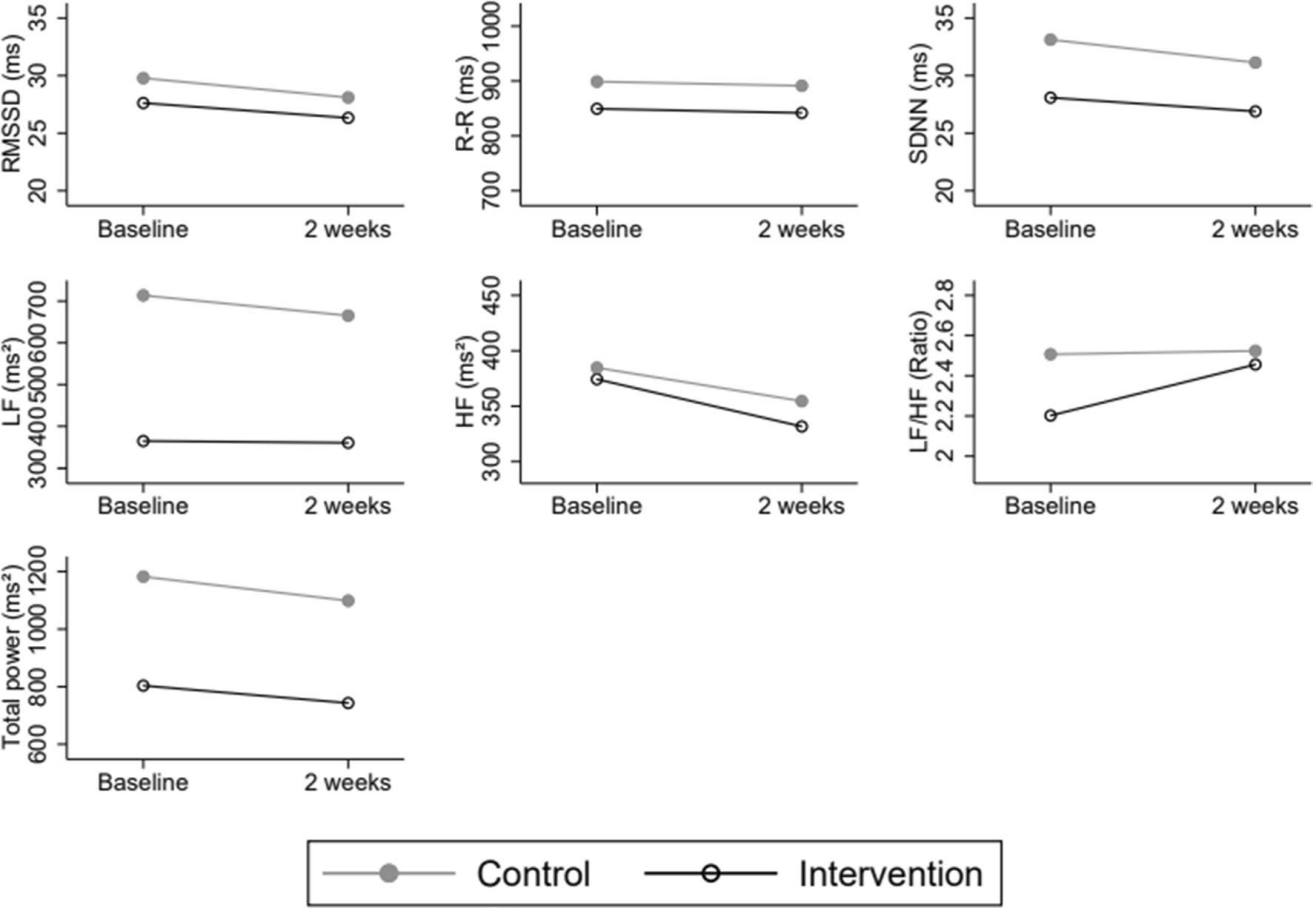
HRV indices. Means for all heart rate variability indices at baseline, 1 week, and 2 weeks modelled using a linear regression model

The interaction effect between time and group is reported in Table 4, with a B-coefficient showing the difference in regression slope between the two groups for each time-point (1 and 2 weeks). The difference is visually available in Fig. 2. No statistically significant group effect was found for any of the HRV indices, with similar results in unadjusted and adjusted models. The adjusted model is found in Additional file 2. Additional details from the analysis are found in Additional files 1 and 2.

**Table 4:** Difference in the regression slope for each time point for intervention and control, control group as reference (n = 123)

Group × time	Treatment effect (unadjusted)			
	B	*P* value	95% CI	
R-R (ms)	0.1	0.997	− 17.2	17.4
RMSSD (ms)	0.4	0.829	− 3.1	3.9
SDNN (ms)	0.8	0.548	− 1.8	3.4
LF (ms^2^)	44.2	0.482	− 79.4	167.8
HF (ms^2^)	− 12.5	0.746	− 88.3	63.3
LF/HF	0.2	0.498	− 0.5	0.9
Total Power (ms^2^)	23.1	0.791	− 148.2	194.4

*R-R* mean R-R interval, *RMSSD* root mean square of successive differences, *SDNN* standard deviation of normal to normal, *LF* low frequency, *HF* high frequency, *LF/HF* LF/HF ratio

The time effect for the total study population is reported in Table 5, with the B-coefficient describing the regression slope for each time point (1 and 2 weeks). There was a slight decrease in all HRV indices over time except for LF/HF. However, only SDNN showed a statistically significant change (B = 1.58, *p* = 0.018), indicating reduced global HRV. Additional details from the analysis are found in Additional file 3.

**Table 5:** Time effect for the total study sample, B indicating the regression line for each time point (n = 123)

R-R	B	*P* value	95% CI	
	− 7.64	0.082	− 16.26	0.98
RMSSD	− 1.48	0.098	− 3.23	0.28
SDNN	− 1.58	0.018	− 2.88	− 0.28
LFms	− 25.62	0.414	− 87.27	36.03
HFms	− 36.75	0.056	− 74.51	1.02
LF/HF	0.14	0.420	− 0.20	0.48
Total power	− 71.94	0.089	− 157.28	13.40

*R-R* mean R-R interval, *RMSSD* root mean square of successive differences, *SDNN* standard deviation of normal to normal, *LF* low frequency, *HF* high frequency, *LF/HF* LF/HF ratio

A per protocol analysis did not change the overall estimates or precision and is therefore not reported here.

### Attrition

Four participants dropped out during the first week. Two of these were not happy about receiving home stretching exercises only, one dropped out due to a change in their work schedule following Covid-19, and one canceled without giving a reason. All dropouts were in the control group. It was also found that two participants had received treatment even though they were part of the control group. They were subsequently moved to the intervention group in the per protocol analysis. One participant reported not to have had NP for longer than 6 months when answering the baseline questionnaire. The reason for this is not clear as all participants were screened prior to the initial clinic visit, which required a 6-month duration of NP. This was identified as a protocol deviation.

The study population showed good adherence to the home exercises. 118 out of 131 at baseline returned the training diary. Of these, 93.7% of the intervention group and 87.9% of the control group performed their home exercises at least 12 out of 14 days as seen in Table 6.

**Table 6:** Adherence to home stretching exercises as reported in exercise diaries

Number of days of having performed stretching exercises (out of 14)	10	11	12	13	14
Intervention, n (%)	4 (6.3)	0 (0.0)	10 (15.9)	10 (15.9)	39 (61.9)
Control, n (%)	0 (0.0)	7 (12.7)	6 (10.9)	6 (10.9)	36 (65.5)

All participants (100%) in the intervention group received manual treatment as defined in this study.

### Adverse reactions

Four intense adverse events (defined by ≥ 8/10 (NRS-11) [71]) were reported in the study by three participants in the intervention group and one in the control group. There were no statistically significant differences in mean adverse reaction between the two groups (*p* > 0.05).

## Discussion

We conducted a randomized clinical study of patients with persistent or recurrent NP to investigate the long-term effect on HRV of SMT and home stretching exercises versus home stretching exercises alone. Our findings indicated no significant effect on a wide range of HRV indices in both the time and frequency domains after 2 weeks of SMT and stretching compared to stretching alone. Nor was any overall improvement in HRV observed across groups. A per-protocol analysis did not significantly change the outcomes. This well-controlled RCT contributes to the literature by investigating the long-term effects of consecutive treatments of SMT and stretching. Previous studies have focused on acute effects during and directly following SMT.

The results may indicate that adding SMT to stretching does not improve HRV. This is at odds with previous research which has found mild to moderate evidence of the acute effect of SMT.

The study also measured changes in pain (NRS-11) between the groups, with no significant observed difference between the groups [61]. Pain (either acute or persistent) is known to be related to reduced HRV [17]. Thus, our results are in line with previous literature because neither pain nor HRV changed differently between the intervention and control groups [17].

Both groups demonstrated a slight worsening of HRV in the whole study sample, which could indicate a shift of the ANS towards sympathetic predominance. However, because the worsening of HRV was only significant for one out of seven HRV indices, it is possible that the interventions had no effect on HRV at all.

A possible explanation of the overall trend in reduced HRV might be related to the participants’ expectations. As part of the test protocol, a conditioned pain modulation (CPM) test was included at the end of each visit. This is further explained in the study protocol [25]. The CPM test is an unpleasant procedure (hand submerged in cold water, 0–2 °C). Even though we were careful about doing this after the HRV measurement, being aware of the painful nature of the CPM test that would come next could have influenced the HRV by increased sympathetic activity [72]. This would not be as evident on the first visit, when the participants had not yet experienced the unpleasant procedure, even though it had been explained to them.

Considerable variability in the individual changes in HRV was seen. This may suggest that responses varied considerably between patients. Whether this was due to individual differences in pain, physiological responses to treatments, or other factors remains unknown but warrants further investigation.

### Methodological considerations

A 2-week intervention period might not have been sufficient to capture changes in HRV among people with persistent or recurrent NP. The length of the intervention period was based on three factors: (1) It is not considered ethical to keep the participants in a treatment group for too long if no improvement is seen [36]. (2) Previous studies have shown a significant effect of SMT on low back pain after 2 weeks [73]. (3) Previous studies have investigated the acute effects of SMT on HRV. Thus, a 2-week treatment period with a total of four treatments was considered long-term in relation to previous research.

The chiropractors were allowed to choose the appropriate SMT procedure for each subject, within the limits of the study, to allow a pragmatic approach. Many of the possible participants would be reluctant to participate if they had to receive a specific passive treatment, i.e. manual treatment to the painful neck itself. Patients' concerns and expectations are often taken into consideration in clinical practice and are one of the three cornerstones of evidence-based medicine [74]. The choice of treatment technique was recorded and will be presented in a separate publication.

### Weaknesses

Because a large part of the study sample was recruited through clinics the participants themselves had chosen, perhaps based on previous contact with the clinic, selection bias may have skewed the results. This was addressed in the study design when clinics were chosen based on, among other things, having multi professional teams. Thus, participants recruited at the clinic would be equally likely to have been in contact with other health care providers at the clinic. This sort of bias is, however, difficult to completely remove from a trial. Selection bias can be assumed to be considerable if the participants had seen the study therapist before. Our data do not demonstrate that this was the case.

It was impossible to blind the chiropractors performing the interventions. This is a possible source of bias because the therapists might favor the intervention group. However, the study was designed to control this by providing written and oral information to clinicians about how to interact with the study participants. Our data do not demonstrate that such bias influenced the results.

HRV typically fluctuates during the day [75]. Due to the study's pragmatic design, it was difficult to book the participants at the same time of the day for each measurement. All participants were booked in during the clinic's opening hours (0700-1600).

The ANS is influenced by internal and external factors such as grief, relationship issues, and other unknown underlying diseases that cannot be controlled for. Also, measurement errors and moderate reliability of HRV could possibly have affected the results. The randomized design should balance such factors between groups. However, it was seen that the intervention group had generally higher HRV than the control group at baseline.

It is possible that persistent or recurrent NP is more resistant to SMT than low back pain. Hence, four treatments over 2 weeks might not have been sufficient to see the same improvements for this patient group as observed for low back pain patients [73]. Long-term follow ups might be needed.

As the sample size was based on a calculation of between group differences, repeated measures might have affected the power of this study, but the effect is unknown.

### Strengths

The main strength of this article is the randomized controlled design.

The study participants did not know what intervention the other group was receiving, and the research assistant and statistician were blinded to group allocation.

Both groups received the same number of treatments and amount of attention from the chiropractors. The control group went through a palpatory examination at each visit, even though treatment was not intended. This was important to balance the contextual effects. The study was pragmatic by nature and mirrored typical treatment strategies.

HRV is recognized as a valid and reliable non-invasive measure of ANS. However, about 40% of a single HRV measurement variance can be explained by the situational effects and person-situation interaction [53]. To address this, a protocol of the procedure was produced and implemented in all participating clinics, before commencing the data collection. The specific measurements and test procedures were also practiced by the two researchers performing them and individually planned for each clinic. The two researchers observed each other to calibrate the measurements and instructions given to patients. This consistency in HRV measurements is considered a major strength of this study. Finally, similar conditions were maintained for all measurements. These include temperature in the room, elimination of disturbing noises, and no alcohol, heavy exercise, or caffeine before the measurements. All these measures were taken to minimize situational effects and person-situation interaction. The number of dropouts was small and adherence to treatment was excellent.

The result from this study indicates that the observed immediate effects of SMT on HRV have no clinical implications over 2 weeks for this patient group.

Future research might examine the relationship between changes in pain and HRV during treatment, include patients with higher pain intensity, or provide a longer treatment period.

## Conclusion

Adding SMT to a 2-week stretching protocol did not result in improved HRV in this well-controlled RCT. Previous findings about the immediate effects on HRV of SMT do not seem to be transferable to a long-term effect, based on the current trial using a longer follow-up time period.

## Supplementary Information

**Additional file 1**. Difference in the regression slope for each time point for intervention and control, control group as reference with all details from the regression model (n = 123) (Unadjusted).

**Additional file 2**. Difference in the regression slope for each time point for intervention and control, control group as reference with all details from the regression model (n = 123) (Adjusted for age, sex, and NRS baseline values).

**Additional file 3**. Time effect for the total study sample, B indicating the regression line for each time point with all details from the regression model (n = 123).

**Additional file 4**. **Unadjusted file 4.** Difference in the regression slope for each time point for intervention and control, control group as reference (n = 123), unadjusted ln values.

**Additional file 5**. Home stretching exercises.

## Data Availability

The data that support the findings of this study are available from Karolinska Institutet. Restrictions apply to the availability of these data, which were used under license for the current study, and so are not publicly available. With the permission of Karolinska Institutet, data are available from the authors upon reasonable request.

## Abbreviations

NP: Neck pain
SMT: Spinal Manipulative Therapy
HRV: Heart Rate Variability
NRS-11: 11-Point numeric rating scale
ANS: Autonomic nervous system
