# Supplementary material for: The effect of spinal manipulative therapy and home stretching exercises on heart rate variability in patients with persistent or recurrent neck pain: a randomized controlled trial
Source: Chiropr Man Therap. 2021 Nov 29;29:48. doi: 10.1186/s12998-021-00406-0 (PMC8628060; doi:10.1186/s12998-021-00406-0)
Supplement: Supplementary file 1 — Additional file 1. Difference in the regression slope for each time point for intervention and control, control group as reference with all details from the regression model (n = 123) (Unadjusted). [file 12998_2021_406_MOESM1_ESM.doc]

|  | B | Std.Error | t | P-value | 95% CI | |
| --- | --- | --- | --- | --- | --- | --- |
| RR GroupxTime | 0.07 | 8.78 | 0.01 | 0.994 | -17.23 | 17.37 |
| RR Time | -7.60 | 6.37 | -1.19 | 0.234 | -20.15 | 4.94 |
| RMSSD GroupxTime | 0.39 | 1.79 | 0.22 | 0.829 | -3.13 | 3.91 |
| RMSSD Time | -1.68 | 1.29 | -1.30 | 0,196 | -4.23 | 0.87 |
| SDNN GroupxTime | 0.80 | 1.32 | 0.60 | 0.548 | -1.81 | 3.40 |
| SDNN Time | -1.99 | 6.37 | -1.19 | 0,039 | -3.88 | -0.10 |
| LFms GroupxTime | 44.19 | 62.73 | 0.70 | 0.482 | -79.41 | 167.80 |
| LFms Time | -48.52 | 45.47 | -1.07 | 0,287 | -138.11 | 41.07 |
| HFms GroupxTime | -12.49 | 38.46 | -0.33 | 0.746 | -88.28 | 63.29 |
| HFms Time | -30.15 | 27.84 | -1.08 | 0.280 | -85.00 | 24.70 |
| LF/HF GroupxTime | 0.24 | 0.35 | 0.68 | 0.498 | -0.45 | 0.93 |
| LF/HF Time | 0.02 | 0.25 | 0.07 | 0.947 | -0.48 | 0.51 |
| Total Power GroupxTime | 23.07 | 86.94 | 0.27 | 0.791 | -148.23 | 194.36 |
| Total Power Time | -83.60 | 63.00 | -1.33 | 0.186 | -207.71 | 40.51 |

Additional file 1. Difference in the regression slope for each time point for intervention and control, control group as reference with all details from the regression model (n=123). (Unadjusted)
