# Supplementary material for: The effect of spinal manipulative therapy and home stretching exercises on heart rate variability in patients with persistent or recurrent neck pain: a randomized controlled trial
Source: Chiropr Man Therap. 2021 Nov 29;29:48. doi: 10.1186/s12998-021-00406-0 (PMC8628060; doi:10.1186/s12998-021-00406-0)
Supplement: Supplementary file 2 — Additional file 2. Difference in the regression slope for each time point for intervention and control, control group as reference with all details from the regression model (n = 123) (Adjusted for age, sex, and NRS baseline values). [file 12998_2021_406_MOESM2_ESM.docx]

Additional file 2. Difference in the regression slope for each time point for intervention and control, control group as reference with all details from the regression model (n=123). (Adjusted for age, sex, and NRS baseline values)

|  | B | Std.Error | t | P-value | 95% CI | |
| --- | --- | --- | --- | --- | --- | --- |
| RR GroupxTime | 1.83 | 8.71 | 0.21 | 0.834 | -15.33 | 18.98 |
| RR Time | -9.55 | 6.31 | -1.51 | 0.132 | -21.98 | 2.89 |
| RMSSD GroupxTime | 0.60 | 1.77 | 0.34 | 0.736 | -2.88 | 4.07 |
| RMSSD Time | -1.83 | 1.28 | -1.43 | 0.153 | -4.34 | 0.68 |
| SDNN GroupxTime | 0.93 | 1.32 | 0.71 | 0.480 | -1.66 | 3.53 |
| SDNN Time | -2.06 | 0.96 | -2.16 | 0.032 | -3.94 | -0.18 |
| LFms GroupxTime | 49.25 | 62.62 | 0.79 | 0.432 | -74.12 | 172.63 |
| LFms Time | -52.38 | 45.39 | -1.15 | 0.250 | -141.81 | 37.05 |
| HFms GroupxTime | -12.06 | 38.41 | -0.31 | 0.754 | -87.74 | 63.62 |
| HFms Time | -29.94 | 27.82 | -1.08 | 0.283 | -84.74 | 24.87 |
| LF/HF GroupxTime | 0.19 | 0.35 | 0.56 | 0.579 | -0.50 | 0.89 |
| LF/HF Time | 0.06 | 0.25 | 0.25 | 0.805 | -0.44 | 0.563 |
| Total Power GroupxTime | 27.94 | 86.87 | 0.32 | 0.748 | -143.20 | 199.09 |
| Total Power Time | -87.02 | 62.98 | -1.38 | 0.168 | -211.10 | 37.06 |
