# Supplementary material for: The effect of spinal manipulative therapy and home stretching exercises on heart rate variability in patients with persistent or recurrent neck pain: a randomized controlled trial
Source: Chiropr Man Therap. 2021 Nov 29;29:48. doi: 10.1186/s12998-021-00406-0 (PMC8628060; doi:10.1186/s12998-021-00406-0)
Supplement: Supplementary file 3 — Additional file 3. Time effect for the total study sample, B indicating the regression line for each time point with all details from the regression model (n = 123). [file 12998_2021_406_MOESM3_ESM.docx]

Additional file 3. Time effect for the total study sample, B indicating the regression line for each time point with all details from the regression model (n=123).

|  | B | Std. Error | t | P-value | 95% CI | |
| --- | --- | --- | --- | --- | --- | --- |
| R-R | -7.64 | 4.37 | -1.75 | 0.082 | -16.26 | 0.98 |
| RMSSD | -1.48 | 0.89 | -1.66 | 0.098 | -3.23 | 0.28 |
| SDNN | -1.58 | 0.66 | -2.39 | 0.018 | -2.88 | -0.28 |
| LFms | -25.62 | 31.29 | 0.82 | 0.414 | -87.27 | 36.03 |
| HFms | -36.75 | 19.17 | -1.92 | 0.056 | -74.51 | 1.02 |
| LF/HF | 0.14 | 0.17 | 0.81 | 0.420 | -0.20 | 0.48 |
| Total Power | -71.94 | 43.31 | -1.66 | 0.089 | -157.28 | 13.40 |
