# Supplementary material for: The effect of spinal manipulative therapy and home stretching exercises on heart rate variability in patients with persistent or recurrent neck pain: a randomized controlled trial
Source: Chiropr Man Therap. 2021 Nov 29;29:48. doi: 10.1186/s12998-021-00406-0 (PMC8628060; doi:10.1186/s12998-021-00406-0)
Supplement: Supplementary file 4 — Additional file 4. Unadjusted file 4. Difference in the regression slope for each time point for intervention and control, control group as reference (n = 123), unadjusted ln values. [file 12998_2021_406_MOESM4_ESM.docx]

Additional file 4. Difference in the regression slope for each time point for intervention and control, control group as reference (n=123), unadjusted ln values.

| **Group x Time** | B | Std.Error | t | P-value | 95% CI |  |
| --- | --- | --- | --- | --- | --- | --- |
| lnR-R (ms) | -0.00 | 0.01 | -0.06 | 0.955 | -0.02 | 0.02 |
| lnRMSSD (ms) | -0.04 | 0.06 | -0.72 | 0.476 | -1.54 | 0.72 |
| lnSDNN (ms) | 0.05 | 0.05 | 0.85 | 0.398 | -0.06 | 0.15 |
| lnLF (ms^2^) | 0.10 | 0.10 | 1.05 | 0.297 | -0.09 | 0.30 |
| lnHF (ms^2^) | -0.03 | 0.10 | -0.27 | 0.790 | -0.23 | 0.18 |
| lnLF/HF | 0.15 | 0.09 | 1.77 | 0.078 | -0.17 | 0.32 |
| lnTotal Power (ms^2^) | 0.04 | 0.09 | 0.46 | 0.649 | -0.14 | 0.22 |
